# Self-reported mandible bracing and teeth clenching are associated with anxiety and depression traits in a group of healthy young individuals

**DOI:** 10.22514/jofph.2024.041

**Published:** 2024-12-12

**Authors:** Ovidiu Ionut Saracutu, Daniele Manfredini, Alessandro Bracci, Edoardo Ferrari Cagidiaco, Marco Ferrari, Anna Colonna

**Affiliations:** ^1^Department of Medical Biotechnologies, School of Dentistry, University of Siena, 53100 Siena, Italy; ^2^Department of Neurosciences, School of Dentistry, University of Padova, 35128 Padova, Italy

**Keywords:** Awake bruxism, Self-report, Psychological status, Anxiety, Depression, STAB

## Abstract

To assess the correlation between awake bruxism (AB) behaviors and psychological status in a group of healthy young adults. Participants were recruited at the University of Siena, Siena, Italy, by advertising the initiative. The reported frequency of AB behaviors was evaluated through the Oral Behavior Checklist (OBC). The 4-item Patient Health Questionnaire-4 (PHQ-4) was adopted to evaluate the participants’ psychological status. Student’s *t*-test was used to detect differences between genders. The Pearson correlation test was performed to assess the correlation between the two questionnaires. Mandible bracing showed the strongest correlation with anxiety and depression traits (*r* = 0.62), followed by teeth clenching (*r* = 0.54). Teeth contact (*r *= 0.33) and teeth grinding (*r *= 0.32) had the lowest level of correlation. In a sample of healthy young individuals, there is a moderate-to-high correlation between the reported teeth clenching and mandible bracing frequency and the degree of anxiety and depression symptoms. Such findings suggest the importance of the psychological assessment in awake bruxers.

## 1. Introduction

In the last decade, the construct of bruxism has been reconceptualized. In the 2013 consensus paper, bruxism has been described as a masticatory muscle activity with two circadian manifestations: awake bruxism (AB) and sleep bruxism (SB) [1]. In a second consensus paper published in 2018 by an international group of experts, awake bruxism has been defined as “*a masticatory muscle activity during wakefulness characterized by clenching and grinding of the teeth and by bracing or thrusting of the mandible and is not a movement disorder in otherwise healthy individuals*” [2]. After the last part of the definition created some concerns in the research community regarding the possibility of considering bruxism a disorder, an explanatory note was published five years after the second consensus paper, clarifying that bruxism is not a disorder per se but, at most, a sign of a co-occurring disease [3]. Within these premises, awake bruxism is nowadays considered an umbrella term for indicating a series of masticatory muscle activities (*i.e.*, teeth contact, mandible bracing, teeth clenching, teeth grinding) that may be a sign or associated with an underlying condition.

Concerning the epidemiology in the general population, the last two systematic reviews on the topic found a prevalence of self-reported AB ranging from 16% to 32% [4, 5]. Data must nonetheless be interpreted with caution since most of the studies are based on a self-reported questionnaire, and among them, the majority assessed AB through one/two single-item questions. Such an assessment method relies on the individual’s capability to recall his/her behaviors in the past months and classifies AB as a black-or-white condition, an approach no longer in line with the current knowledge [6].

Recently, there has been a fundamental breakthrough in the field of bruxism assessment [7, 8], which led to the development of the first non-stackable, multidimensional evaluation system of bruxism, the STAB (Standardized Tool for the Assessment of Bruxism) [9]. The STAB can guide clinicians and researchers in assessing bruxism. After the publication of such a comprehensive tool, a screening instrument was quickly developed (Bruxscreen), which is easy to administer in large-scale epidemiological research projects [10]. The Bruxscreen contains a part concerning the self-reported assessment of some AB behaviors (*i.e.*, teeth contact, mandible bracing, teeth clenching, teeth grinding) based on a 5-point Likert scale questions (*i.e.*, never, sometimes, regularly, often, always, do not know), taken from the Oral Behavior Checklist (OBC) [11].

As for the etiology, despite the fact that peripheral factors (*i.e.*, features of dental occlusion) were in the past claimed to be related to bruxism, several papers have shown the absence of such association [12, 13]. A paradigm shift toward centrally-mediated factors characterized the new millennium, with much focus on the psychological sphere [14, 15, 16]. Particularly distressing scenarios like the COVID-19 pandemic, due to their psychological burden, were responsible for an increase in AB behaviors [17, 18]. A study performed on temporomandibular disorders (TMD) patients found a significant association between the frequency of AB behaviors and the degree of psychological impairment [19]. AB activity was also shown to be higher in post-traumatic stress disorder (PTSD) patients than in the general population [20]. Another study performed in a sample of college preparatory students assessed the AB behaviors frequency in relation to psychological factors such as depression, anxiety, and stress and found a statistically significant association [21]. Similarly, a study performed on Israeli undergraduate dentistry students showed a significant correlation between the Ecologically Momentary Assessment (EMA) of AB and depression [22]. Moreover, self-reported AB was found to be correlated with anxiety and depression in a sample of patients undergoing orthodontic treatment [23]. Instrumental assessment of AB confirmed a probable link between increased masticatory muscle activity and anxiety traits [24]. However, no existing study has tried to correlate specific AB behaviors with the level of psychological distress in a healthy population. Only one recent retrospective investigation found a dose-response association between non-functional waking-state oral behaviors and psychological distress [25].

Within these premises, this study aims to assess the correlation of specific self-reported AB behaviors included in the STAB and BruxScreen evaluation (*i.e.*, teeth contact, mandible bracing, teeth clenching, teeth grinding) with the psychological status assessed through the 4-item Patient Health Questionnaire 4 (PHQ-4), which is also included in the STAB [26].

## 2. Materials and methods

### 2.1 Participants recruitment

Participants were recruited, without gender or ethnic restriction, at the University of Siena, Siena, Italy, by advertising the initiative. The inclusion criterion was a good general health without any neurological, systemic, autoimmune or oral diseases. Exclusion criteria were any ongoing medical or dental treatment or past treatments for AB and temporomandibular disorders (TMDs). The TMD Pain screener was administered to rule out TMD patients [27]. All participants received verbal and written information about the intent of the investigation.

### 2.2 Study design

After being enrolled in the study and signing the informed consent, volunteers attended a one-hour seminar with the leading investigator and the study supervisor. Participants listened to an explanatory lesson concerning the new definition of awake bruxism and oral behaviors that can occur during wakefulness. At the end of the seminar, participants received an anonymous questionnaire containing in the following order: the TMD Pain Screener [27], the four awake bruxism items of the OBC (Items A2.1, A2.2, A2.3, A2.4 of the STAB) [11], and the PHQ-4 (Item B1.1 of the STAB) [26].

### 2.3 Awake bruxism assessment

The first part of the questionnaire contained four questions from the Oral Behavior Checklist concerning awake bruxism behaviors frequency in the last month [11]:

i. How often do you grind your teeth together during waking hours, based on the last month? (Item A2.1 of the STAB);

ii. How often do you clench your teeth together during waking hours, based on the last month? (Item A2.2 of the STAB);

iii. How often do you press, touch, or hold your teeth together other than while eating (that is, contact between upper and lower teeth), based on the last month? (Item A2.3 of the STAB);

iv. How often do you hold, tighten, or tense your muscles without clenching or bringing teeth together, based on the last month? (Item A2.4 of the STAB).

For each question, individuals are requested to indicate the frequency of the behavior, using a 5-point Likert scale as follows: “none of the time” (0), “a little of the time” (1), “some of the time” (2), “most of the time” (3), “all of the time” (4).

### 2.4 Psychological assessment

The PHQ-4 (Item B1.1 of STAB) [26] was used to screen for potential anxiety and depression. The questionnaire is an ultra-brief self-report scale based on four questions, two related to anxiety and two regarding depression:

“*Over the last two weeks, how often have you been bothered by the following problems?*”

i. Feeling nervous, anxious or on edge;

ii. Not being able to stop or control worrying;

iii. Feeling down, depressed or hopeless;

iv. Little interest or pleasure in doing things.

For each item, the subject is requested to indicate how often they experience each sensation: Not at all = 0, Several days = 1, More than half the days = 2, Nearly every day = 3. The total score can range from 0 to 12 and can be rated as normal (0–2), mild (3–5), moderate (6–8), and severe (9–12). A total score ≥3 for the first two questions suggests anxiety, while a total score ≥3 for the last two questions suggests depression.

### 2.5 Statistical analysis

Statistical analysis was performed with Microsoft Office Excel 2021 (Los Angeles, CA, USA). The Pearson test was used to assess the correlation between the OBC frequency of each AB behavior and the PHQ-4 questionnaire. The student’s *t*-test was performed to detect differences between genders. A *p*-value < 0.05 was considered statistically significant.

## 3. Results

Of the 110 volunteers who were recruited and filled out the TMD pain screener, 10 were excluded due to TMDs. All the remaining 100 met the inclusion criteria and were included in the study (31 males and 69 females, mean age 22.5 years ± 2.5, range 19–29) (Fig. 1).

**Fig. 1.** Flowchart of participants recruitment. TMD: temporomandibular disorders.

Table 1 indicates the OBC frequency of the four awake bruxism activities investigated in this study (*i.e.*, teeth contact, mandible bracing, teeth clenching, and teeth grinding). “None of the time” was the most frequently reported frequency (134 times), followed in order by a “little of the time” (97), “some of the time” (95), “most of the time” (62), and “all of the time” (12). No participant reported an “all of the time” frequency for mandible bracing and teeth grinding, and among all the masticatory activities, teeth grinding was the least reported behavior. No statistically significant difference was present in the OBC status for males and females (*p* > 0.05).

**Table 1.** Number of times each condition was indicated (OBC).

	None of the time = 0	A little of the time = 1	Some of the time = 2	Most of the time = 3	All of the time = 4
Teeth Contact	26	17	30	16	11
Mandible Bracing	30	29	25	16	0
Teeth Clenching	17	30	27	25	1
Teeth Grinding	61	21	13	5	0
Total	134	97	95	62	12

The PHQ-4 scores for the study sample are reported in Table 2. More than half of the participants did not report any symptoms of anxiety and/or depression (65%). Conversely, the remaining 35% of the participants had a PHQ-4 score ≥3, with only 2 participants reporting severe anxiety and depression.

**Table 2.** PHQ-4 status of the considered sample.

Depression and anxiety status	PHQ-4
0–2 = normal	65% (N = 65)
3–5 = mild	14% (N = 14)
6–9 = moderate	19% (N = 19)
10–12 = severe	2% (N = 2)

*PHQ-4: The Patient Health Questionnaire-4.*

The results of the Pearson correlation test between the frequency of each specific awake bruxism behavior and the PHQ-4 scores are reported in Table 3. Mandible bracing showed the strongest positive correlation with anxiety and depression traits, followed by teeth clenching. Teeth grinding had a moderate correlation. Teeth contact and teeth grinding had a lower degree of correlation. All pairwise correlation tests were statistically significant.

**Table 3.** Pearson correlation test between the four AB behaviors and the PHQ-4.

OBC/PHQ-4 Correlation	*r*	*p*-value
Teeth Contact	0.33	0.013
Mandible Bracing	0.62	<0.001
Teeth Clenching	0.54	<0.001
Teeth Grinding	0.32	<0.001

*OBC: Oral Behavior Checklist; PHQ-4: 4-item Patient Health Questionnaire-4.*

## 4. Discussion

Concerning the etiology of bruxism, scientific evidence shows that there has been a shift from occlusal-centered theories to a biopsychosocial model [13]. The psychological assessment [9] can be a potentially good predictor of bruxism [28] and orofacial pain [29].

Based on these premises, this cross-sectional study aimed to measure the degree of correlation between each single behavior frequency belonging to the AB spectrum and the level of psychological distress in a sample of healthy volunteers using a questionnaire containing the OBC and the PHQ-4.

The OBC questionnaire is part of the STAB [9]. Compared to dichotomous classification, which investigates just the presence or absence of awake bruxism, this tool, based on a Likert scale, allows clinicians to perform a quantitative and qualitative evaluation of each behavior in the last month. Since questionnaires are the easiest method to collect data on bruxism, most of the studies on AB prevalence are currently based on self-report [4]. For this purpose, it is worth mentioning that the main limitation of self-reported AB is the recall bias; patients might not be able to recall precisely the type and the frequency of the reported oral behaviors [22]. However, the OBC still represents a valid and reliable first screening tool to quickly obtain a general overview of the possible AB behaviors frequency and recognize the patient’s self-perception. For the purposes of this study, the functional activities of the OBC were not included since it was demonstrated that they are not detrimental to the stomatognathic system [30].

When it comes to assessing anxiety and depression status, the PHQ-4 is a widely accepted tool in the field of orofacial pain (Item B1.1 of STAB) [31]. This questionnaire has been rigorously validated in a large sample of 5030 participants [26]. Its ultra-brief scale equips clinicians with a straightforward method to screen anxiety and depression using just four questions. The resulting score is directly proportional to the severity of the symptoms, making it a valuable tool in the clinical setting.

The main result of this study is that among all AB activities, mandible bracing, a low-level, long-lasting contraction of a masticatory muscle without teeth contact, has the strongest correlation with anxiety and depression scores.

Under these premises, it is possible to hypothesize that mandible bracing is likely to occur more frequently in subjects affected by anxiety and depression, symptoms that are common findings in TMD patients [32]. Thus, mandible bracing could be a possible link between psychological factors and TMDs, which can be indeed exacerbated by the severity of the psychological distress. In support of this hypothesis, a recent case-control study performed with the EMA approach showed that TMD patients experience a much higher frequency of mandible bracing during wakefulness than healthy individuals [33]. Despite a certain amount of physiological AB expected in pain-free symptoms [34], what seems to determine the persistence of TMD pain is the frequency of mandible bracing. As a proof of concept, an experimental study demonstrated that induced mandible bracing, a 5-minute protocol of 5 seconds of master muscle contraction, followed by 1 second of rest, can evoke pain, soreness, fatigue, and stiffness. In the experimental group, the discomfort disappeared after 24 hours because of masseter muscle rest [35]. In TMD patients, the prolonged mandible bracing activity and the lack of rest could instead lead to the persistence of pain.

Teeth clenching frequency was also found to have a moderate correlation with anxiety and depression, although to a lesser extent than bracing. Moreover, in a similar experimental protocol, induced teeth clenching [36] was also demonstrated to lead to myofascial pain [37]. Instead, teeth grinding was poorly associated with psychological distress as well as the least reported masticatory muscle activity in the OBC questionnaire, thus indirectly supporting the validity of the assumptions behind the expansion of bruxism definition to the broader spectrum of non-grinding activities. Also, other studies based on EMA concluded that teeth grinding during wakefulness was almost never reported in healthy study populations [21, 38, 39, 40, 41, 42, 43, 44], orthodontic patients [45], and TMD patients [33]. Due to all this evidence, it is possible to hypothesize that teeth grinding might not be a determinant behavior in the AB spectrum of activities nor a clinically relevant condition during wakefulness.

The study findings align with previous papers describing the link between psychological factors and AB frequency [13, 46, 47, 48, 49, 50]. Studies showed that AB behaviors can be interpreted as a reaction, during wakefulness, to stressful daily events. The frequency of such behaviors can vary according to the individual stress-coping ability and sensitivity to stress [12]. Personality traits such as neuroticism and extraversion have also been linked to bruxism [51]. From a neurobiological perspective, the impact of the psyche is explained by a lack of balance in the dopaminergic motor pathways [52]. Emotional tension could force the subject to respond to stressful stimuli by contracting the masticatory muscles for a prolonged time [53].

This study presents some limitations. First, no clinical examination was performed to look for signs of ongoing AB activity. Second, the PHQ-4 is a tool to screen for symptoms of anxiety and depression, but no specific diagnosis has been performed on the participants. Nevertheless, large epidemiological studies showed a high criterion validity of the PHQ-4. In a large sample of 1052 patients, the PHQ-4 subscales had high sensitivity, ranging from 0.9 to 0.88 for the PHQ-2 and generalized anxiety disorder-2 (GAD-2) and a clinically acceptable specificity of 0.61 for both subscales [54]. Moreover, a recent cross-sectional investigation on more than five thousand subjects unveiled a very high value for the PHQ-4’s internal consistency of 0.92, making it a suitable questionnaire for screening anxiety and depression in studies with large samples, such as the current study.

To improve the study design, further research should try correlating the psychological traits with AB, adopting instrumental devices capable of quantifying and discriminating among the different oral behaviors, such as surface electromyography (EMG) [55]. EMG instruments are indeed already on the market. However, the lack of standardized guidelines on electromyographic trace interpretation and algorithms capable of discriminating the different types of masseter muscle activity [9] pose some limits for EMG usage in clinical settings to evaluate AB [56].

## 5. Conclusions

In a sample of healthy subjects, there is a moderate-to-high correlation between teeth clenching and mandible bracing frequency and the degree of anxiety and depression symptoms. Such correlation is lower for teeth contact and teeth grinding.
